# Detection of Chemical Warfare Agents with a Miniaturized High-Performance Drift Tube Ion Mobility Spectrometer Using High-Energetic Photons for Ionization

**DOI:** 10.1021/acs.analchem.2c03422

**Published:** 2022-10-27

**Authors:** André Ahrens, Maria Allers, Henrike Bock, Moritz Hitzemann, Arne Ficks, Stefan Zimmermann

**Affiliations:** † Leibniz University Hannover, Institute of Electrical Engineering and Measurement Technology, Department of Sensors and Measurement Technology, Appelstr. 9A, 30167Hannover, Germany; ‡ Bundeswehr Research Institute for Protective Technologies and CBRN Protection, Humboldtstraße 100, Munster29633, Germany

## Abstract

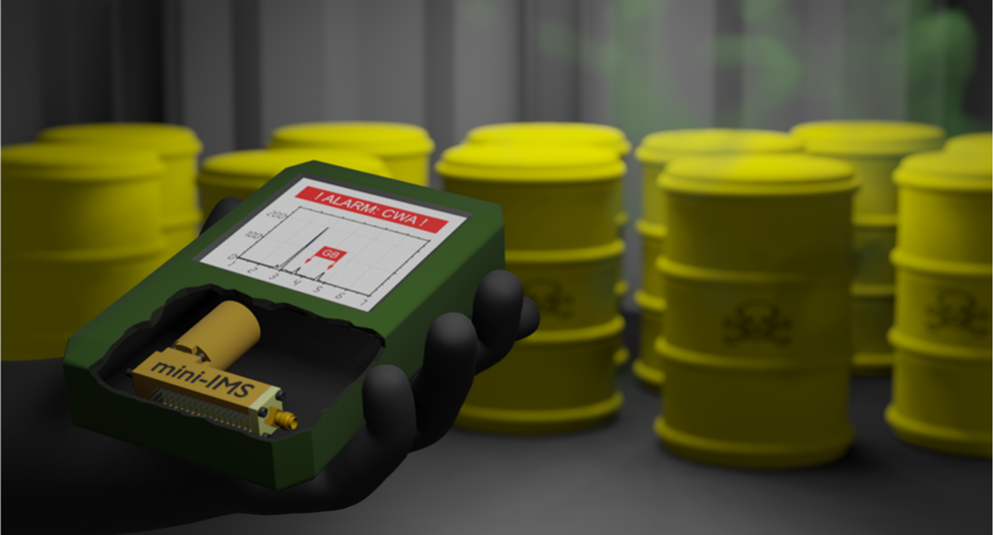

A growing demand for low-cost gas sensors capable of detecting the smallest amounts of highly toxic substances in air, including chemical warfare agents (CWAs) and toxic industrial chemicals (TICs), has emerged in recent years. Ion mobility spectrometers (IMS) are particularly suitable for this application due to their high sensitivity and fast response times. In view of the preferred mobile use of such devices, miniaturized ion drift tubes are required as the core of IMS-based lightweight, low-cost, hand-held gas detectors. Thus, we evaluate the suitability of a miniaturized ion mobility spectrometer featuring an ion drift tube length of just 40 mm and a high resolving power of *R*_p_ = 60 for the detection of various CWAs, such as nerve agents sarin (GB), tabun (GA), soman (GD), and cyclosarin (GF), as well as the blister agent sulfur mustard (HD), the blood agent hydrogen cyanide (AC) and the choking agent chlorine (CL). We report on the limits of detection reaching minimum concentration levels of, for instance, 29 ppt_v_ for sarin (GB) within an averaging time of only 1 s. Furthermore, we investigate the effects of precursors, simulants, and other common interfering substances on false positive alarms.

## Introduction

In recent years, there has been an increasing demand for miniaturized, low-cost sensors capable of detecting the smallest amounts of highly toxic airborne chemicals. Sensors complying with these attributes are integrated into lightweight hand-held gas detectors that are built to warn military forces or civilian responders in the event of a premeditated or accidental release of chemical warfare agents (CWAs) or toxic industrial chemicals (TICs).^1,2^ In order to provide a timely warning to the operator, such chemical detection equipment must possess detection limits well below immediately life- and health-threatening concentrations. Furthermore, these devices ideally have short response times, generate easy-to-interpret data, and are largely unaffected by environmental factors.^3,4^ Many commercially available hand-held gas detectors (e.g., LCD 3.3,^5^ RAID-M100Plus,^6^ GDA-P,^7^ ChemProX,^8^ and so forth) are based on ion mobility spectrometry, as this technology largely meets the aforementioned requirements. Comprehensive general information on ion mobility spectrometry is available in dedicated publications by Eiceman et al.^9^ and Borsdorf et al.^10^

Generally, ion mobility spectrometry technology relies on the selective movement of ions opposing a neutral buffer gas under the influence of an electric field, leading to the separation of different ion species. At low electric fields, the ion’s drift velocity *v*_D_ is proportional to the ion mobility *K* at a given electric field strength *E* (eq 1). Low-field conditions typically result from reduced electric fields between 2 and 10 Td, depending on the ion species.^11^
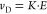
1

The mobility measurement using a traditional drift tube IMS is initiated with the injection of an ion packet into the drift region of the drift tube. The field-driven ion motion along the tube leads to the specific separation of different ion species before reaching the detector at the end of the drift tube. An ion mobility spectrum is subsequently obtained by plotting the ion current at the detector against the elapsed flight time. For the described setup, the ion mobility *K* can be derived from the length *L* of the drift tube, the drift voltage *U*_D_, and the drift time *t*_D_ (eq 2).
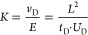
2

The ion mobility *K* is typically normalized using the standard temperature *T*_0_ = 273.15 K and the standard pressure *p*_0_ = 1013.25 mbar in order to account for the influence of a changing neutral gas molecule density at varying pressures *p* and temperatures *T*. The resulting normalized value is commonly referred to as the reduced ion mobility *K*_0_ (eq 3). It is worth noting that this normalization does not consider different drift gas humidities or other parameters affecting the ion mobility. Thus, caution is advised when comparing reduced ion mobilities measured with different devices under different conditions.
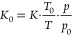
3

The analytical performance of an IMS device comprises sensitivity that is directly observed as the signal-to-noise ratio and resolving power *R*_p_ (eq 4). A high resolving power *R*_p_ is reached when the full width at half maximum *w*_0.5_ of a measured ion peak is small in relation to the corresponding drift time *t*_D_.
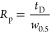
4

Hand-held gas detectors necessarily require a miniaturization of the whole detection system, including the IMS drift tube but also other peripheral components such as power supply, filters and pumps. Here, we focus on the IMS drift tube itself. The development of a compact high-performance drift tube is quite challenging since the analytical performance strongly depends on its geometric dimensions.^12,13^ For example, the compact drift tube (about 30 mm drift length) integrated into LCD 3.3 achieves a resolving power of *R*_*p*_ = 15.^14,15^ At this moderate resolving power, only ion species with significantly different reduced ion mobilities can be separated. Hence, commercially available hand-held gas detectors based on IMS generally show many false positives due to their limited analytical performance.^3,14,16−19^ The occurrence of false positives may have serious consequences in theater of operations, especially if operators are unaware of the existing technical limitations, and even if operators are aware of the aforementioned limitations, alarms may be interpreted as false (acclimatization effect caused by a high false positive rate) and therefore wrongfully ignored. Accordingly, it is of uttermost interest to minimize the false positive rate of IMS-based hand-held gas detectors, which requires compact drift tube designs to incorporate sufficient analytical performance.

Recently, we presented a miniaturized high-performance drift tube IMS manufactured from polyether ether ketone, stripes of stainless-steel foil, and printed circuit boards.^20^ The drift tube design, coined mini-IMS, provides a high resolving power of *R*_p_ = 60 at a drift length of just 40 mm. In this work, we subjected a drift tube IMS of this type to CWAs for the first time and subsequently evaluated the capabilities and limitations for future use as a hand-held gas detector. The measurements with highly toxic CWAs were performed in dedicated laboratories at the Bundeswehr Research Institute for Protective Technologies and CBRN Protection (WIS), Munster, Germany. A number of representative live agents, specifically nerve agents sarin (GB), tabun (GA), soman (GD), and cyclosarin (GF); blister agent sulfur mustard (HD); blood agent hydrogen cyanide (AC); and choking agent chlorine (CL) were tested. For evaluation purposes, the limits of detection (LOD) and the reduced ion mobilities (*K*_0_) of these substances were determined. Furthermore, we estimated the extent of false positive alarms by examining common interfering substances.

## Experimental Section

### Miniaturized High-Performance Drift Tube Ion Mobility Spectrometer

**Figure 1:**
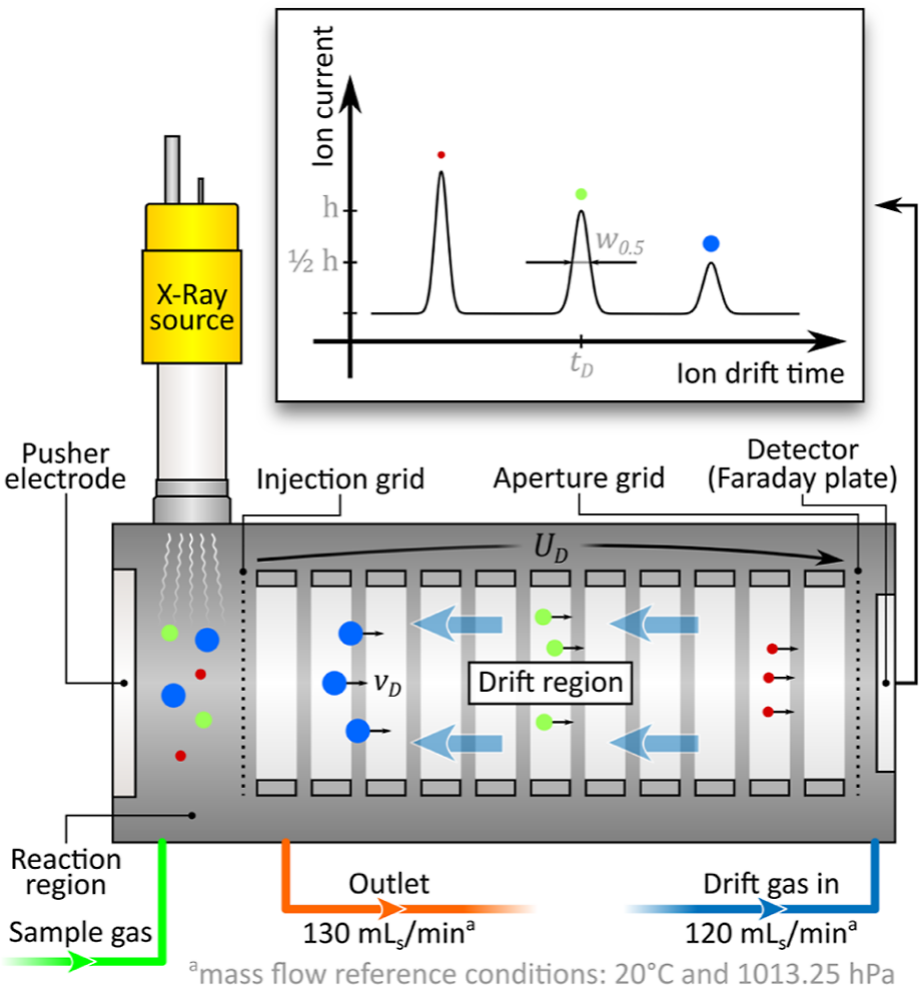
Schematic of the miniaturized high-performance drift tube IMS used in this work.

**Figure 2:**
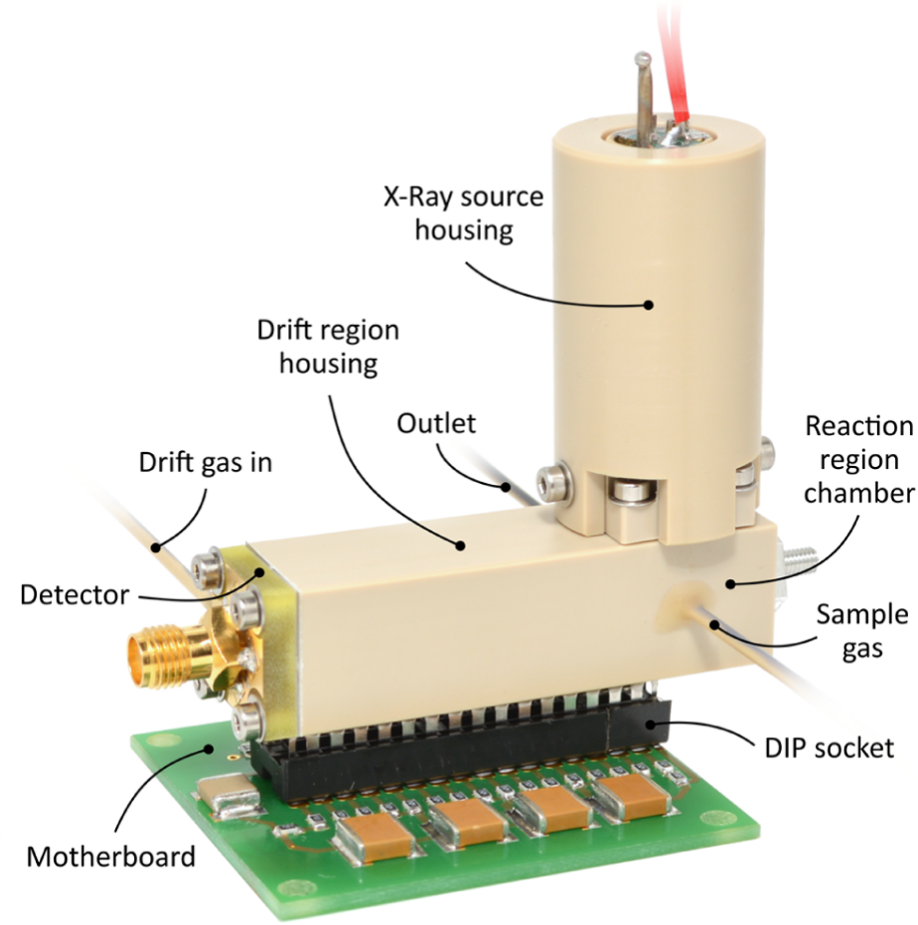
Photo of the miniaturized high-performance drift tube IMS with just 40 mm drift length. Adapted with permission from ref (20). Copyright 2019, The Author(s).

A detailed description of the utilized miniaturized high-performance drift tube IMS and its application for the detection of volatile TICs can be found in earlier publications.^20,21^Figure 1 shows the schematic of the measurement setup, including the drift tube design and the basic operation principle. Additionally, Figure 2 shows a photo of the miniaturized high-performance IMS drift tube. The default operating parameters used in this study are summarized in Table 1. In simple terms, the ion drift tube consists of three main parts: a reaction region, a drift region, and a shielded Faraday plate as the ion detector. In the reaction region, ion generation is initiated by high energetic photons emitted from an X-ray source (Model XRT-50-2-Rh-0.6-125, Newton Scientific Inc., Cambridge, Massachusetts, USA) that is placed orthogonally with respect to the direction of ion movement.^22^ A field-switching ion shutter is used to push ions from the reaction region into the drift region.^23^ Subsequently, in the drift region, ions move along the electric field of 625 V/cm toward the shielded Faraday plate. The ion drift tube can be operated in either positive or negative ion mode by simple inversion of the applied voltages. The sample gas is introduced directly into the reaction region, while the drift gas is led into the drift region near the detector. Synthetic air with less than 2 ppm_v_ of H_2_O and less than 1 ppm_v_ of CO_2_ is used as the drift gas at a flow rate of 120 mL_s_/min (standard milliliter per minute, mass flow at reference conditions 20 °C and 1013.25 mbar) controlled by a mass flow controller (MFC) (F-201CV-200-RAD-33-V, Bronkhorst Deutschland Nord GmbH, Kamen, Germany). The sample gas flow is adjusted indirectly by controlling the total gas flow at the outlet. For this reason, the outlet is equipped with a MFC (F-201CV-200-RAD-33-V, Bronkhorst Deutschland Nord GmbH, Kamen, Germany) and a pump (NMP015KPDC-B4, KNF Neuberger GmbH, Freiburg, Germany) to create sufficient vacuum. The total gas flow at the outlet is set to 130 mL_s_/min to eliminate memory effects and a possible cross-contamination of the sample gas. Temperature data for *K*_0_ determination are collected by monitoring the drift tube housing temperature using a temperature sensor (LM95071CIMF/NOPB, Texas Instruments, Dallas, Texas, USA). The pressure inside the IMS drift tube is determined by means of a pressure sensor (AMS 5812 - 0150 - B, AMSYS GmbH & Co. KG, Mainz, Germany) that is located directly at the IMS outlet. During operation, the temperature and pressure typically vary in the range of 32.0–33.5 °C and 1005–1020 mbar, respectively.

**Table 1:** Operating Parameters of the Miniaturized High-Performance Drift Tube IMS

temperature *T*	32.0–33.5 °C
pressure *p*	1005–1020 mbar
drift gas	synthetic air (< 2 ppm_v_ H_2_O, < 1 ppm_v_ CO_2_)
drift gas flow	120 mL_s_/min^a^
sample gas	purified air (about 3% RH and 400 ppm_v_ CO_2_) containing specified traces of CWA
sample gas flow	10 mL_s_/min^a^
drift region length *L*	40 mm
drift region voltage *U*_D_	2.5 kV (pos.)
	–2.5 kV (neg.)
reaction region length	2 mm
reaction region voltage	550 V (pos.)
	–550 V (neg.)
ionization source	X-rays
	filament current: 630 mA
	acceleration voltage: –3.4 kV
injection time	100 μs
repetition rate	40 Hz

a mL_s_/min: standard milliliter per minute, mass flow at reference conditions 20 °C and 1013.25 mbar.

### Chemicals and Gases

The liquid nerve agents sarin (GB), tabun (GA), soman (GD), and cyclosarin (GF), as well as the blister agent sulfur mustard (HD), were synthesized at WIS. The purities of the agents were determined by nuclear magnetic resonance spectroscopy and were above 90%. Gas cylinders containing 10 ppm_v_ of the gaseous blood agent hydrogen cyanide (AC) or the choking agent chlorine (CL) were purchased from Dräger Safety, Lübeck, Germany (part numbers 6810642 and 6812106).

### Sample Gas Preparation

**Figure 3:**
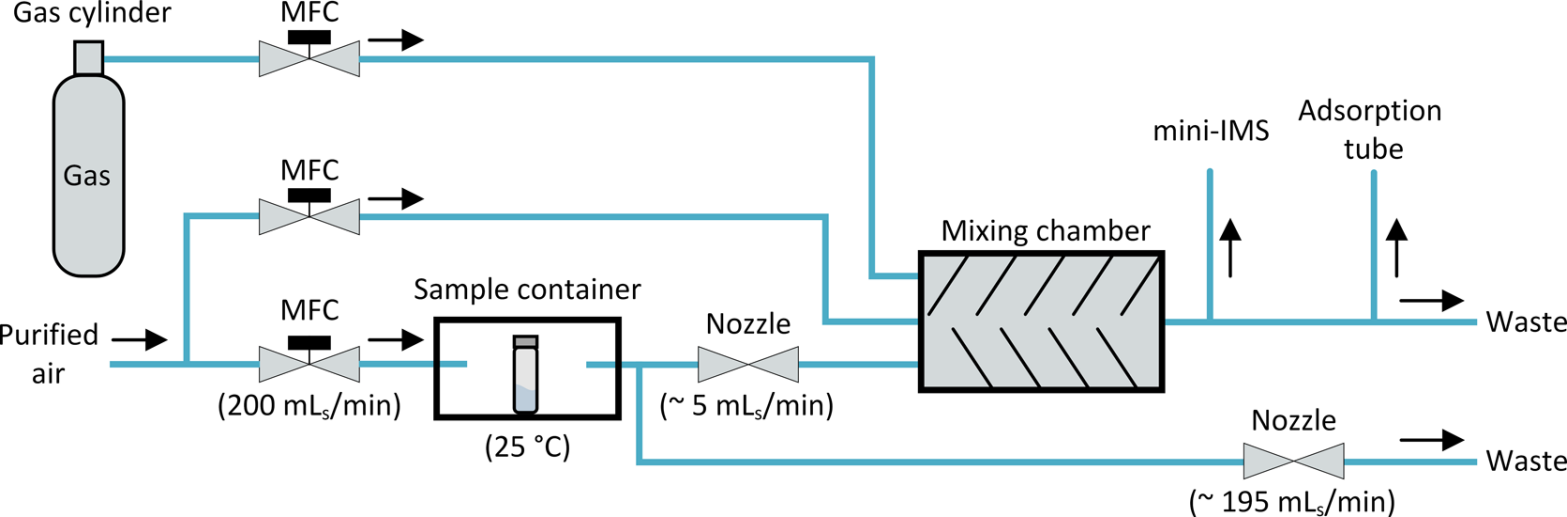
Gas mixing system for sample gas preparation, including a test gas cylinder, a mixing chamber, MFCs, and nozzles.

The sample gas was prepared in the gas mixing system shown in Figure 3. Sample gas containing GB, GA, GD, GF, and HD was generated by the permeation technique. The agents were sealed in polyethylene plastic tubes and placed in a sample container at a constant temperature of 25 °C. Small amounts of agent permeating through the tube wall evaporate into the atmosphere and are diluted by an adjustable flow of purified air that passes through the sample container. Via two fixed flow resistors (nozzles), a small amount of this gas flow is directed into a gas mixing chamber for optional further dilution by the addition of purified air. To determine the resulting test gas concentration, Tenax TA adsorption tubes were loaded downstream of the mixing chamber and analyzed by calibrated thermodesorption–gas chromatography–mass spectrometry (TD–GC–MS). For calibration, Tenax TA adsorption tubes were spiked with 5, 10, 20, 30, 40, and 50 ng of each agent. The sample gas containing AC and CL was generated by diluting the air from gas cylinders with purified air. The resulting test gas concentration was calculated from the mixing ratio of the gases.

## Results and Discussion

**Figure 4:**
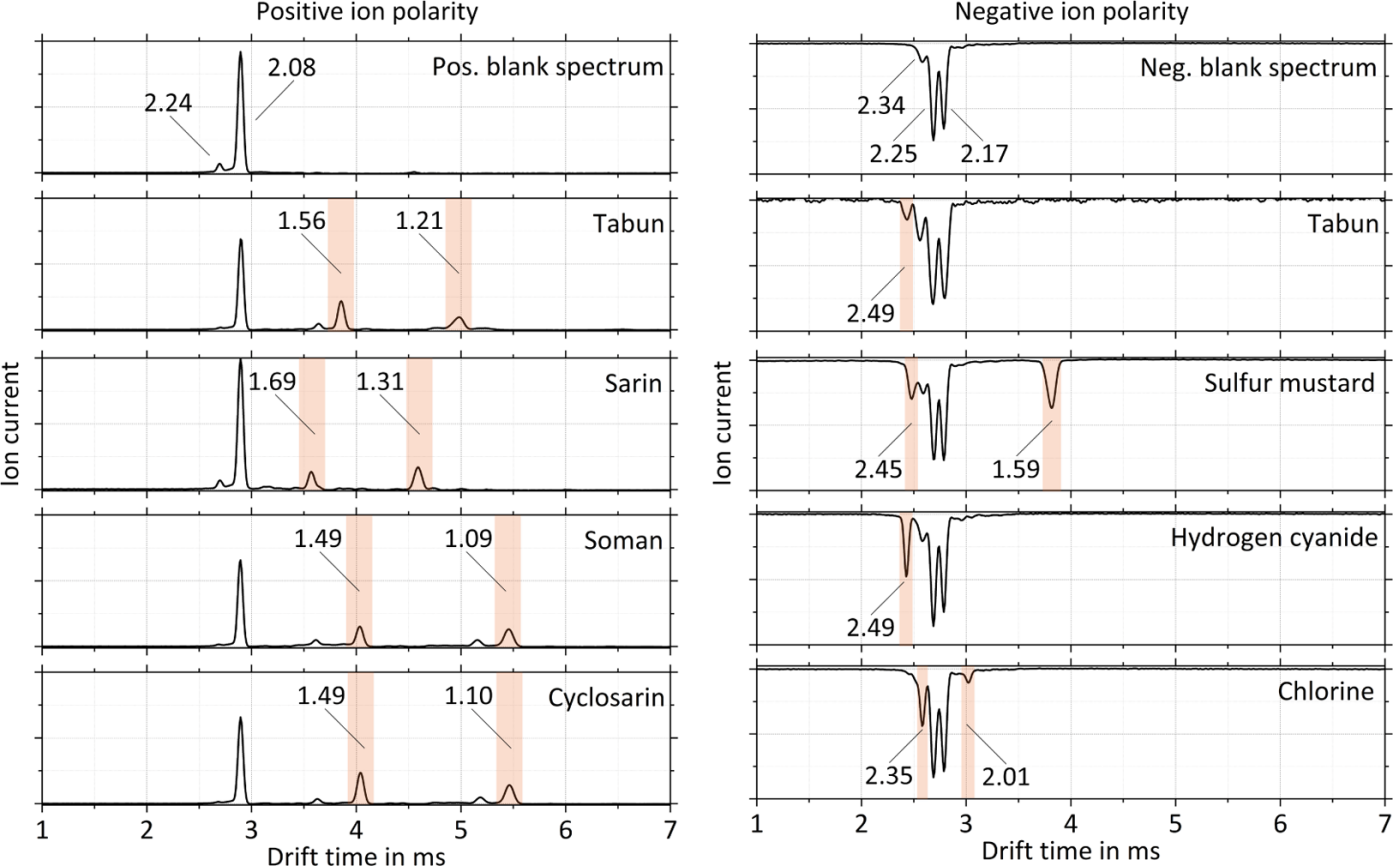
Measured ion mobility spectra of nerve agents tabun (2 ppb_v_), sarin (1 ppb_v_), soman (2 ppb_v_), and cyclosarin (3 ppb_v_); blister agent sulfur mustard (6 ppb_v_); blood agent hydrogen cyanide (7 ppb_v_); and choking agent chlorine (5 ppb_v_). The product ion peaks are highlighted and labeled with the corresponding reduced ion mobility K_0_ in cm^2^/Vs. The operating parameters are given in Table 1.

In order to evaluate the analytical performance of the miniaturized high-performance drift tube IMS with respect to the detection of CWAs, ion mobility spectra of nerve agents sarin (GB), tabun (GA), soman (GD), and cyclosarin (GF); blister agent sulfur mustard (HD); blood agent hydrogen cyanide (AC); and choking agent chlorine (CL) were recorded. Figure 4 shows the measured ion mobility spectra of these substances. The product ion peaks are highlighted and labeled with the corresponding reduced ion mobility *K*_0_. The measured reduced ion mobilities *K*_0_ of the product ions are also summarized in Table 2.

**Table 2:** Reduced Ion Mobilities *K*_0_ in cm^2^/Vs of CWAs Measured in This Work and by Other Studies^a^

		without dopant							NH_3_ doped	
reference		this work	WIS	Sohn et al.^25^	manufacturer^33^	Yamaguchi et al.^34^	Yang et al.^35^	Seto et al.^29^	Satoh et al.^14^	Bocos-Bintintan et al.^15^
device		mini-IMS	RAID-1	RAID-1	RAID-1	SABRE 4000	GDA-2	custom device	LCD 3.3	LCD 3.2E
sarin (GB)	peak 1	1.688 (+)	1.69 (+)	1.68 (+)	1.62 (+)		1.68 (+)		1.56 (+)	
	peak 2	1.311 (+)	1.28 (+)	1.28 (+)	1.22 (+)	1.24 (+)	1.28 (+)		1.25 (+)	
tabun (GA)	peak 1	1.563 (+)	1.54 (+)	1.58 (+)	1.51 (+)				1.44 (+)	
	peak 2	1.209 (+)	1.15 (+)	1.18 (+)	1.06 (+)	1.18 (+)			1.25 (+)	
	peak 3	2.491 (−)	2.45 (−)	3.09 (−)	2.44 (−)				2.39 (−)	
soman (GD)	peak 1	1.486 (+)	1.86 (+)		1.51 (+)		1.81 (+)		1.35 (+)	
	peak 2	1.093 (+)	1.27 (+)		1.06 (+)	1.12 (+)	1.06 (+)		1.04 (+)	
cyclosarin (GF)	peak 1	1.490 (+)	1.50 (+)						1.36 (+)	
	peak 2	1.103 (+)	1.07 (+)			1.13 (+)			1.04 (+)	
sulfur mustard (HD)	peak 1	2.446 (−)	2.41 (−)	2.37 (−)	2.40 (−)			2.35 (−)		
	deak 2	1.587 (−)	1.56 (−)	1.56 (−)	1.55 (−)			1.58 (−)	1.47 (−)	
hydrogen cyanide (AC)	peak 1	2.492 (−)	2.53 (−)	2.50 (−)	2.44 (−)	2.45(−)		2.47 (−)	2.33 (−)	2.38 (−)
chlorine (CL)	peak 1	2.346 (−)				2.30 (−)		2.33 (−)	2.13 (−)	
	peak 2	2.007 (−)								

a The ion polarity is given in brackets.*K*_0_ values of this work are determined based on 1000 averaged ion mobility spectra.

As expected, organophosphorus nerve agents such as sarin, tabun, soman, and cyclosarin have high proton affinities and are therefore readily protonated in the reaction region of the miniaturized high-performance drift tube IMS.^24^ In the positive ion mode, these substances give rise to two separate peaks that are attributed to the hydrated protonated monomer [M·H^+^(H_2_O)_*n*_] and the proton bound dimer [M_2_H^+^].^14,24−26^ Among the four nerve agents studied here, only tabun can be detected in the negative ion mode. The negative ion mobility spectra of both tabun and hydrogen cyanide contain a peak at *K*_0_ = 2.49 cm^2^/Vs, and hydrogen cyanide is a known degradation product of tabun.^27^ The observed peak can therefore be attributed to hydrated cyanide anions [CN^–^(H_2_O)_*n*_]. Regarding the nerve agent ion mobility spectra of soman and cyclosarin, it is worth noting that the respective product ions (both monomer and dimer) exhibit very similar reduced ion mobilities. Thus, most commercial IMS devices are typically programmed to display a combined GD/GF alarm.^14^ Even at an increased resolving power of *R*_p_ = 60 reached with the miniaturized high-performance drift tube IMS, it is impossible to clearly distinguish the two substances.

In contrast to nerve agents sarin, tabun, soman, and cyclosarin, the blister agent sulfur mustard; blood agent hydrogen cyanide; and choking agent chlorine are only detected in the negative ion mode. Sulfur mustard shows two product ion peaks in the ion mobility spectrum, the first of which (*K*_0_ = 2.45 cm^2^/Vs) is attributed to hydrated hydrogen chloride anions [HCl^–^(H_2_O)_*n*_] resulting from a dissociative ionization of sulfur mustard, while the second peak (*K*_0_ = 1.59 cm^2^/Vs) represents the actual product ion of sulfur mustard [HD·O_2_^–^(H_2_O)_*n*_].^28,29^ As mentioned above, the spectrum of hydrogen cyanide contains only the product ion peak of the hydrated cyanide anions [CN^–^(H_2_O)_*n*_] (*K*_0_ = 2.49 cm^2^/Vs).^29^ Chlorine most probably forms hydrated chlorine anions [Cl_2_^–^(H_2_O)_*n*_] that give rise to the first product ion peak (*K*_0_ = 2.35 cm^2^/Vs) in the spectrum.^29,30^ At high chlorine concentrations, a second product ion peak (*K*_0_ = 2.01 cm^2^/Vs) appears that, in turn, may be attributed to a dimer ion.^30^

In Table 2, the measured reduced ion mobilities *K*_0_ of the CWAs are summarized and compared to values from the literature. It is striking that in some cases the reported reduced ion mobilities *K*_0_ differ significantly from each other. As mentioned above, smaller variations in the reduced ion mobility can be explained by different measurement conditions. Generally, the normalization of the ion mobility regarding temperature and pressure suppresses the influence of a changing number of neutral gas molecules per volume in the drift tube. Nevertheless, changes in temperature or humidity may still lead to a minor shift in reduced ion mobility if cluster chemistry is affected by these parameters.^31,32^ The reported variations in the reduced ion mobility values of soman, however, seem too large to be caused by these effects. From previous measurements at WIS, it is known that the ion mobility spectrum of soman strongly depends on the purity of the substance used. We assume that, in the case of soman, some of the reported literature data on reduced ion mobilities actually originates from impurities, such as related precursors or degradation products. Excluding the mentioned outliers, the measured reduced ion mobilities *K*_0_ using the miniaturized high-performance drift tube IMS are generally in agreement with those data stated in the literature.

**Figure 5:**
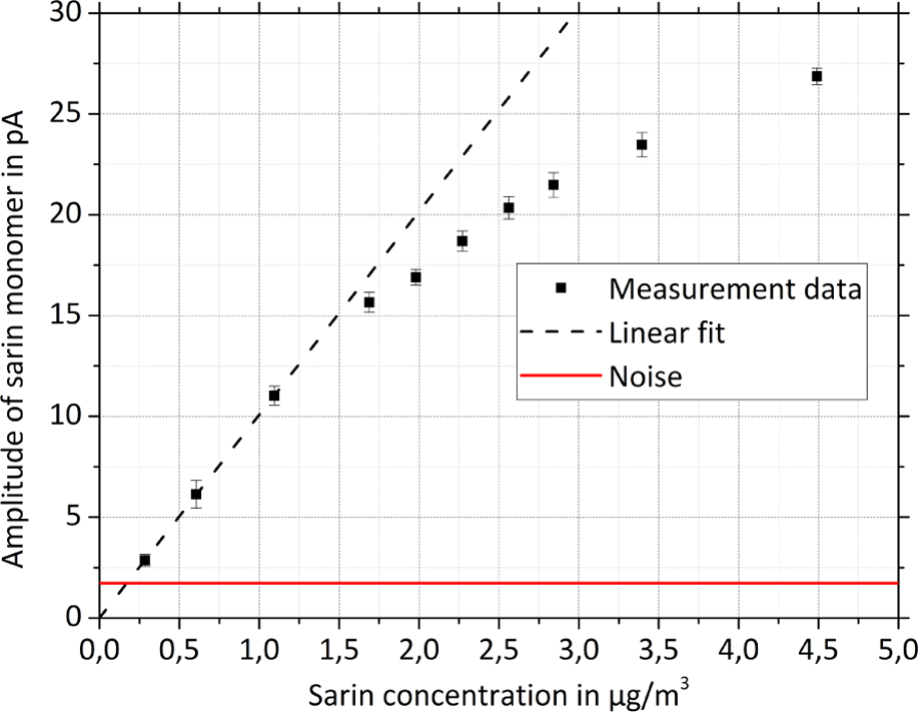
Calibration curve of the sarin monomer at a sample gas humidity of 3% RH. The black squares are measured data points with respective standard deviation of *n* = 10 measurements with 1 s of averaging time each. The dashed black line represents a linear fit through the three data points at low concentration levels, and the solid red line indicates 3 times the standard deviation of the noise at zero concentration. Operating parameters of the miniaturized high-performance drift tube IMS are given in Table 1.

The sensitivity of the miniaturized high-performance drift tube IMS was investigated in a series of measurements. In order to determine the LODs for CWAs, the amplitude of the main product ion peak in the ion mobility spectrum was recorded while varying the concentration of the respective CWA. The resulting calibration curve is exemplarily shown for sarin in Figure 5. The LOD is defined as the concentration that generates a signal amplitude, which is equal to 3 times the standard deviation σ of the noise at zero concentration. The standard deviation σ of the noise at zero concentration was determined using an averaging time of 1 s. As shown in Figure 5, the miniaturized high-performance drift tube IMS reaches an LOD of 0.17 μg/m^3^ for sarin. The determined LODs for other CWAs are summarized in Table 3. In addition to the LODs, the resolving power *R*_p_ is often used as an analytical performance factor. As an example, we have determined the resolving power of the sarin peaks, which is *R*_p_ = 60. Since the miniaturized high-performance drift tube IMS uses a field switching ion shutter, this resolving power *R*_p_ can be approximately transferred to other ion species since the ion mobility *K* of this shutter type is nearly independent of the optimal drift voltage and the maximum resolving power.^36^

**Table 3:** LODs for CWAs Measured with the Miniaturized High-Performance Drift Tube IMS; for Comparison, LODs from the Literature and the 10 min Marginal and 10 min Negligible MEGs^37^ Are Given

		LOD				MEG	
	peak (***K***_0_)	this work	Sohn et al.^25^	manufacturer^33^	Seto et al.^29^	10 min marginal	10 min negligible
sarin (GB)	1.688 cm^2^/Vs (+)	0.17 μg/m^3^(29 ppt_v_)	11.7 μg/m^3^	5 μg/m^3^		140 μg/m^3^	6.9 μg/m^3^
tabun (GA)	1.563 cm^2^/Vs (+)	0.16 μg/m^3^(24 ppt_v_)	6.7 μg/m^3^	5 μg/m^3^		140 μg/m^3^	6.9 μg/m^3^
soman (GD)	1.486 cm^2^/Vs (+)	0.28 μg/m^3^(37 ppt_v_)		5 μg/m^3^		61 μg/m^3^	3.5 μg/m^3^
cyclosarin (GF)	1.490 cm^2^/Vs (+)	0.40 μg/m^3^(53 ppt_v_)				57 μg/m^3^	3.5 μg/m^3^
sulfur mustard (HD)	2.446 cm^2^/Vs (−)	1.09 μg/m^3^(165 ppt_v_)	<33 μg/m^3^	20 μg/m^3^	13000 μg/m^3^	1200 μg/m^3^	400 μg/m^3^
hydrogen cyanide (AC)	2.492 cm^2^/Vs (−)	<1.1 μg/m^3^(<1 ppb_v_)	<11 μg/m^3^	200 μg/m^3^	57 μg/m^3^	19000 μg/m^3^	2800 μg/m^3^
chlorine (CL)	2.346 cm^2^/Vs (−)	<30 μg/m^3^(<10 ppb_v_)		5 μg/m^3^	600 μg/m^3^	8100 μg/m^3^	1500 μg/m^3^

A comparison of the LODs obtained using the miniaturized high-performance drift tube IMS and values from literature as well as the military exposure guidelines (MEG)^37^ in Table 3 reveals the excellent sensitivity of the studied system. The miniaturized high-performance drift tube IMS reaches LODs far better than the 10 min marginal MEG for all CWAs. Even more impressively, LODs are also significantly better than the 10 min negligible MEG. However, it is worth mentioning that the miniaturized high-performance drift tube IMS uses a direct inlet, whereas devices for field use often suffer from decreased sensitivity due to their required inlet systems.

We complete our study by examining precursors and/or simulants of CWAs as well as common interfering substances in order to estimate the extent of false positive alarms. As explained above, several factors affect the reduced ion mobility *K*_0_ and usually lead to variations of approximately ±0.02 cm^2^/Vs or ±2% in the *K*_0_ value of IMS-based instruments.^38^ Due to these variations, wide detection windows are required to minimize the probability of excluding any compounds of interest (false negatives). However, wide detection windows are a source of decreased selectivity as interfering compounds with similar *K*_0_ values may be falsely assigned as target compounds (false positives). In this work, *K*_0_ has been determined by using eqs 2 and 3. Another approach to reduce measurement errors is to measure the *K*_0_ value of a standard which has nearly constant reduced ion mobility independent of the factors mentioned above. Using such a standard, a better comparability of *K*_0_ values can be achieved if, based on the standard, an instrument factor is determined, which is subsequently used to calculate unknown *K*_0_ values. Further information on this method can be found in Hauck et al.^38^ Independent of the method, in order to narrow the detection window, it is essential to determine the reduced ion mobility with a high degree of reproducibility.

**Figure 6:**
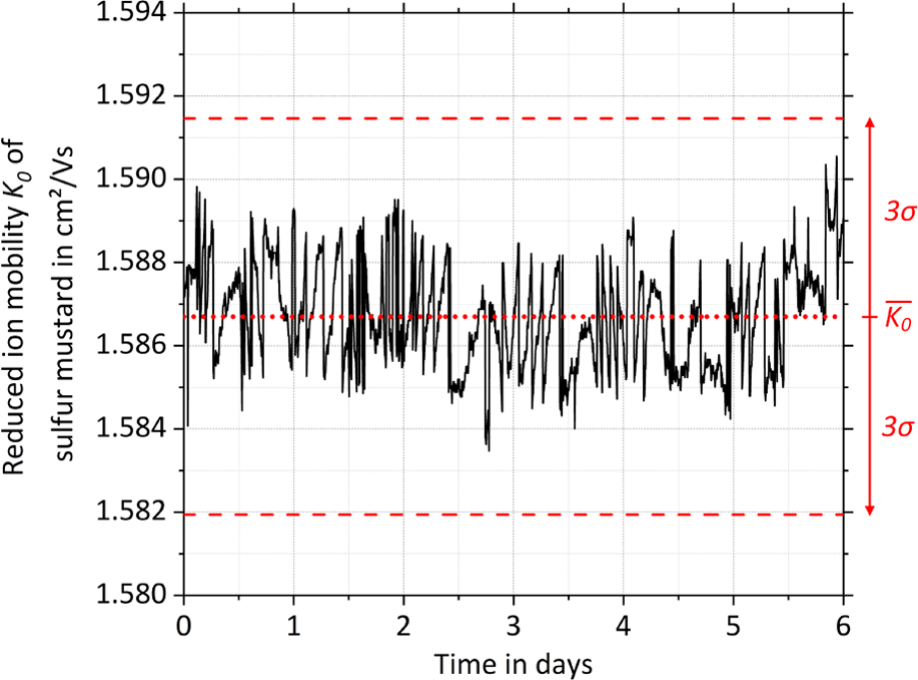
Measured reduced ion mobility *K*_0_ (solid black line) of sulfur mustard over a period of 6 days. The average reduced ion mobility *K*_0_ (dotted red line) ± the 3-fold standard deviation 3σ (dashed red line) is indicated. The 3-fold standard deviation corresponds to a mobility variation of ±0.3%.

For example, in Figure 6, the measured reduced ion mobility of sulfur mustard is plotted over a period of 6 days. During this period, the reduced ion mobility fluctuates around an average value of 1.587 cm^2^/Vs with a 3-fold standard deviation of 0.005 cm^2^/Vs. For the miniaturized high-performance drift tube IMS, we were therefore able to use a detection window width of just ±0.005 cm^2^/Vs or ±0.3%. It is worth noting that the ion mobility fluctuation will increase if the drift gas is provided by a purge loop rather than an external source, since the pump and filters are additional sources of error.

In Table 4, the measured reduced ion mobilities *K*_0_ of several precursors and/or simulants of CWAs as well as common interfering substances are summarized. A false positive CWA alarm is assumed if the measured reduced ion mobilities are within a range of ±0.3% with regard to the reduced ion mobilities of the CWAs in Table 2. As shown in Table 4, only triethylphosphate (TEP) and gasoline vapor may cause false CWA alarms “GA” and “GB”, respectively.

**Table 4:** Reduced Ion Mobilities *K*_0_ in cm^2^/Vs of Precursors and/or Simulants of CWAs as Well as Common Interfering Substances Measured with the Miniaturized High-Performance Drift Tube IMS

	*K*_0_ in cm^2^/Vs	false positives when using the miniaturized high-performance drift tube IMS
Precursor and/or simulants		
TMP	1.764 (+), 1.363 (+)	none
TEP	1.559 (+), 1.230 (+)	GA
2-mercaptoethanol	1.785 (+), 1.600 (+), 1.892 (−)	none
dipropylene glycol monomethyl ether	1.653 (+), 1.223 (+)	none
dimethyl methylphosphonate	1.804 (+), 1.410 (+)	none
diethyl methylphosphonate	1.648 (+), 1.220 (+)	none
diisopropyl methylphosphonate	1.527 (+), 1.092 (+)	none
methyl salicylate	1.503 (−), 1.709 (+)	none
Interfering substances		
acetic acid	1.989 (−), 1.671 (−)	none
triethylamine	1.881 (+), 1.500 (+)	none
*N*,*N*-dimethylformamide	2.019 (+), 1.663 (+)	none
methylisoketone	1.739 (+), 1.391 (+)	none
gasoline vapor	1.838 (+), 1.762 (+), 1.690 (+)	GB
AFFF (firefighting foam)	1.534 (+), 1.113 (+)	none
JP8 (jet propellant)	1.705 (+), 1.387 (+)	none
diethyltoluamide (insect repellent)	1.895 (+), 1.672 (+)	none
eucalyptol (eucalyptus oil)	1.555 (+), 1.191 (+), 1.153 (+)	none

However, it is worth mentioning that despite reproducible *K*_0_ values and narrow detection windows, false negatives can occur due to competing ionization processes in the IMS reaction region when an interfering substance suppresses the ionization of a CWA. This is independent of ion mobilities and is instead a question of ion chemistry, that is, among other things, a matter of proton and electron affinities. A possible solution to this problem would be the temporal pre-separation of complex gas mixtures using fast GCs, for example, which would, however, lead to an undesired increase in response times.

## Conclusions

In this work, the capabilities and limitations of a miniaturized high-performance drift tube IMS were evaluated for its use in hand-held CWA gas detectors. The spectrometer was examined using live agents sarin (GB), tabun (GA), soman (GD), cyclosarin (GF), sulfur mustard (HD), hydrogen cyanide (AC), and chlorine (CL), as well as several interfering substances. Compared to other drift tube IMS devices of similar size, the miniaturized high-performance drift tube IMS exhibits an exceptionally high analytical performance. A particular highlight is its sensitivity for CWAs with LODs in the double-digit ppt_v_ range, well below the 10 min negligible MEG. Furthermore, the high resolving power of *R*_p_ = 60 in combination with the high reproducibility leads to comparatively few false positive alarms. In summary, the miniaturized high-performance drift tube IMS offers significant technical improvements and can therefore be highly recommended for use in hand-held CWA gas detectors.
